# Health Sector Initiatives for Disaster Risk Management in Ethiopia: A Narrative Review 

**DOI:** 10.1371/currents.dis.949664319ad451313b499f9c90cd9c0f

**Published:** 2014-04-01

**Authors:** Luche Tadesse, Ali Ardalan

**Affiliations:** Department of Disaster Public Health, School of Public Health, Tehran University of Medical Sciences, Tehran, Iran; Private Consultant, Addis Ababa, Ethiopia; Department of Disaster & Emergency Health, Iran's National Institute of Health Research; Department of Disaster Public Health, School of Public Health, Tehran University of Medical Sciences, Tehran, Iran; Harvard Humanitarian Initiative, Harvard University

## Abstract

Background: Natural and man-made disasters are prevailing in Ethiopia mainly due to drought, floods, landslides, earthquake, volcanic eruptions, and disease epidemics. Few studies so far have critically reviewed about medical responses to disasters and little information exists pertaining to the initiatives being undertaken by health sector from the perspective of basic disaster management cycle. This article aimed to review emergency health responses to disasters and other related interventions which have been undertaken in the health sector. Methods: Relevant documents were identified by searches in the websites of different sectors in Ethiopian and international non-governmental organizations and United Nations agencies. Using selected keywords, articles were also searched in the data bases of Medline, CINAHL, Scopus, and Google Scholar. In addition, pertinent articles from non-indexed journals were referred to. Results: Disaster management system in Ethiopia focused on response, recovery, and rehabilitation from 1974 to 1988; while the period between 1988 and 1993 marked the transition phase towards a more comprehensive approach. Theoretically, from 1993 onwards, the disaster management system has fully integrated the mitigation, prevention, and preparedness phases into already existing response and recovery approach, particularly for drought. This policy has changed the emergency response practices and the health sector has taken some initiatives in the area of emergency health care. Hence, drought early warning system, therapeutic feeding program in hospitals, health centers and posts in drought prone areas to manage promptly acute malnutrition cases have all been put in place. In addition, public health disease emergencies have been responded to at all levels of health care system. Conclusions: Emergency health responses to drought and its ramifications such as acute malnutrition and epidemics have become more comprehensive in the context of basic disaster management phases; and impacts of drought and epidemics seem to be declining. However, the remaining challenge is to address disasters arising from other hazards such as flooding in terms of mitigation, prevention, preparedness and integrating them in the health care system. Key Words: Disaster, Emergency Health, Health System, Ethiopia

## BACKGROUND

**Disasters in Ethiopia**

Natural and man-made disasters and public health emergencies are quite common in Ethiopia due to drought, flood, earthquake, volcanic-eruptions, epidemics of communicable diseases, dry and wet mass movement 1 , 2 , 3 , 4 . Among the natural disasters, drought is the most devastating one since it has been historically common in the northern part of Ethiopia and recently affecting central, eastern, southern parts of the country as well. It has been documented that drought occurred from 1969 to 2011 in different annual intervals affecting millions of people in each event 3 , 5 . Since 1974, the occurrence of drought has become more frequent, severe in magnitude, and has been causing loss of human lives and property damage. The most severe events were in the year 1974, 1983, 1987, 1989, 2003, 2005, 2008, and 2009 2 , 5 ,7 . The population in drought areas are vulnerable because of their reliance on seasonal rain-fall for their livelihood and hence affecting them in myriad ways-ranging from severe malnutrition to deaths and loss of livestock and agricultural related properties. For instance, in 1974 an estimated 200, 000 people died due to drought in the northern part of the country 3 , since then the short and long-term effects of drought have been persisting in various forms including severe acute and chronic malnutrition, and associated morbidities and mortalities due to measles, respiratory infections (mainly pneumonia), diarrhea and dysentery. In 1985/86, a study conducted in Borana (southern part of Ethiopia), which is one of the drought prone areas, revealed that high level of malnutrition and wasting was associated with child mortality 8_. _In the same year (in February 1985), another study was conducted in emergency camps of Ethiopian drought refugees in Sudan reported that a crude mortality rate of 8.9 per 10, 000 per day, and the mortality had been the highest among children under five years of age (22 per 10, 000 per day) 9 . On the other hand, a study carried out from December 1999 to July 2000 in Somali region of Ethiopia, another prominent drought affected area, reported a crude mortality rate of 3.2 per 10000 per day (with 95% Confidence Interval/CI 2.4-3.8/10000 per day) among children 6 months to 14 years of age 10 . Following the 2003 drought, the vulnerable people were resettled in various parts of the country under resettlement programs. For example, from the eastern Hararge (eastern part of Ethiopia) vulnerable people were made to resettle in the western part of the country. A nutritional survey was carried out in 2004 among the resettled and vulnerable population in western part of Ethiopia. According to this survey, prevalence of global acute malnutrition was 9.8% (95% CI 7.1-12.5), prevalence of severe acute malnutrition was 1.93% (95% CI 0.68-3.18%), and the under-five mortality rate was 2.65 per 10,000 per day 11 . As can be analyzed from the above studies, the mortality rates had been by far higher than the threshold set for declaring an emergency for each age-specific category of mortality and children under-five years have been found to be the most vulnerable and victims of drought. Though timing in any survey is very important in order to objectively establish the association of malnutrition and drought, national surveys such as Demographic and Health Surveys (DHS) demonstrate high levels of wasting and stunting among children. For example according to DHS conducted in 2011, 10% and 44 % of children had been wasted and stunted, respectively 12 . Hence, drought has been the leading disaster in Ethiopia besides other emerging and frequent hazards.

Floods are the second natural disaster in Ethiopia. They have been occurring more frequently and affecting the country almost every year between 1993 and 2013 2 ,5 ,13_. _Basing on limited available data, between 1980 and 2010 an estimated 45 flooding events had happened in different parts of the country 2 . The main types of floods in Ethiopia are flash and riverine (overflow of rivers and inundating the nearby regions). The most catastrophic floods documented have been in 2006, which affected many regions of Ethiopia and an estimate of over 600 people were killed and more than 500,000 people affected 2 ,5 ,13 ,14_. _Limited studies have been carried out on how flood events affect human health in the country. A qualitative study conducted in western part of Ethiopia reported that injuries, lack of pure water supply, hygiene and sanitation services, and malaria epidemic outbreaks are the major consequences of floods 15 .

Different epidemic occurrence is another disaster causing mortality and morbidity in the country 1 ,2 ,5 . Among the epidemics include measles, dysentery, meningococcal meningitis, anthrax, influenza A and B, relapsing fever and yellow fever are the major, as commonly reported by the government 1 ,16_. _Some of these epidemics are associated with natural disasters such as drought and floods. As stated above, during drought malnutrition has been common and the fact that malnutrition has immuno-compromising effect on an individual, in most instances it is associated with measles, dysentery and other communicable diseases. Though few studies have been carried out in Ethiopia following flooding events, post flood communicable disease outbreaks such as dysentery and malaria are also quite common 15 .

Other disasters arising from earthquake and volcanic eruptions 1 ,2 ,5 , landslides or land movement 1 ,2 ,5 ,17 are also happening in Ethiopia being more peculiar to the rift valley region. They occasionally cause loss of human lives and livestock, and property damage. Landslides are mainly triggered by deforestation, heavy rain fall, intervention of the land for road construction and construction of dams 17 ,18 . The effects of these hazards on human health have not been adequately documented. However, it has been estimated that their effects have so for not been as catastrophic as those of drought’s and floods’ 5 .

Disaster management system in Ethiopia dates back from the 1974 drought which claimed thousands of lives 3 . Since then, the disaster management policies, strategies, structures have been changing over time by subsequent Ethiopian governments 4 . Parallel with change in disaster risk management system, the health care system of Ethiopia also evolved over time from traditional curative approach to focus on accessing primary health care services 5 **.**Few studies thus far critically reviewed the evolution of disaster management system in Ethiopia. There has been little information about initiatives taken by health sector to address the prevailing natural and man-made disasters as well as public health emergencies. A paucity of information exists as to the main focus of Ethiopian government regarding disaster management. Hence, this paper is set to bridge the above information gap.

## OBJECTIVE

The primary aim of this article is to review the emergency healthcare interventions undertaken in the Ethiopian health system in relation to disaster response and to assess the available initiatives of health sector regarding disaster risk management from the perspective of basic disaster management cycle that entails mitigation, prevention, preparedness, response and recovery.

## METHODS

The analysis about the health sector initiatives for disaster risk management done based on the policy and strategic documents related to disaster risk management of successive regimes of Ethiopia, and based on available data and studies conducted on disasters and emergencies in the country. Therefore, relevant documents were identified by searches in the websites of Ethiopian Federal Ministry of Health, Disaster Risk Management and Food Security Sector, Central Statistics Agency of Ethiopia, The United Nations Office for Disaster Risk Reductions , Center for Research on the Epidemiology of Disasters, Relief and Prevention Webs. Data bases of Medline, CINAHL, Scopus, and Google Scholar were searched for articles. The key words used were: disaster OR emergency AND Ethiopia; health care OR medical care AND Ethiopia; drought OR flood OR epidemic OR famine OR malnutrition AND Ethiopia. All articles related to Ethiopia were considered for review without applying any exclusion criteria on year of publication. First, articles were identified by their titles, and then the abstracts were read to select the articles relevant to the subject matter. In addition, articles from non-indexed journals and book chapters were also referred to.

## RESULTS AND DISCUSSION

**1 EMERGENCY HEALTH CARE IN ETHIOPIA**

**1.1 Disaster Risk Management System and General Emergency Response**

Historically, the 1974 drought was one of the worst disasters that resulted in loss of an estimated 200, 000 human lives, caused huge outbreaks of diseases, loss of live-stock, and made massive internal displacement. To coordinate the relief and rehabilitation efforts during the event, Ethiopian government established Relief and Rehabilitation Commission (RRC). Its main responsibilities were to mobilize resources from national and international sources and organize human power to deploy to the affected areas. This drought did not only lead to establishment of RRC but also triggered the down fall of the government of Haileslaise and reinstatement of military junta (the Derg regime) 3 ,4 . After ten years, in 1983/1984 another drought occurred which affected millions of Ethiopian population 2 ,3 ,5 .

The mandate of established RRC was limited to coordination of relief. Till 1989 it had not transformed itself to an agency that could implement disaster prevention and preparedness programs. Due to absence of adequate preparedness measures, a number of human lives were lost even in the two severe droughts of 1983/1984 and 1989 which could have been avoided 4 ,19 ,20 . During those droughts, the measures undertaken were to appeal for international aid, relief programs such as provision of food and medical services to the affected people, and resettlement of affected population to other regions within the country 3 ,21 ,22 . As some of these actions were unplanned, the health conditions of famine affected people were further exacerbated due to reactive response to disaster. For example, the resettlement action was taken without preparation and the disease outbreaks among the settlers were higher than the local people, and health services were inadequately accessed by the resettled people in various regions 23 . Moreover, responses to disaster were not timely during the 1983/1984 drought, like the former one where government failed to readily admit and declare the existence of drought in the country since the government was at war with rebels 3 . It was the international media that uncovered the situation in the country 22 and some aid agencies or individual donors decided to channel the fund through rebels so as to support the affected population in the rebel held area. However, there was an allegation that instead the rebels diverted some aid to purchase weapons. This attests to the ineffective relief coordination and aid due to absence of strong preventive and preparedness system in place 21 ,22 . In this period and afterwards, there was lack of vital “lifesaving emergency health interventions” such as treatment and prevention of measles, malaria treatment and provision of pure water supply 24 . Furthermore, some of the preparedness initiatives such as the early warning system though had started before 1977, they were criticized for being ineffective for a number of reasons. Firstly, the early warning system was centralized and involvement of the local government and community was limited. Second, the indicators used had been only addressing the food security and rainfall status, while overlooking the comprehensive dimension that purported to include such as malnutrition and diseases outbreaks status in the community. Third, the early warning system as a whole failed to give the necessary information before the occurrence of drought of 1983/84 and 1989/2000 25 . While the initially used early warning system helped for the subsequent development of relatively comprehensive and informative early warning system for the drought, the other hazards were not incorporated and there was no other preparedness measure being under taken for any other hazard or disaster both at policy level and in practice till 1989. Hence, the year between 1974 and 1989 marked different events of severe drought disasters that claimed thousands of lives due to lack of effective preventive, preparedness, and mitigation programs within disaster management system of the country.

In May 1991, the military regime was replaced by the current government and the already formulated policies and strategies of the disaster mitigation, prevention, preparedness, response, recovery and rehabilitation were re-stated and adopted as “National Policy on Disaster Prevention and Management” by the transitional government of Ethiopia in 1993 26 . However, the previously existing structure of disaster management body was changed. Consequently in 1993, the coordinating body known as “National Disaster Prevention and Preparedness Committee (NDPPC)” was established at national level to oversee the overall disaster management programs in the country 27 . Furthermore, in 1995 the RRC was renamed Disaster Prevention and Preparedness Commission (DPPC) to coordinate nationally the whole program of disaster management from disaster mitigation, prevention, preparedness, response, to recovery and rehabilitation 4 . More recently, DPPC has been named Disaster Risk Management and Food Security Sector and it is operating under supervision of Ministry of Agriculture 28 . The structure of disaster management that exists at national level involving all line ministries has been replicated at regional, zonal, and district levels.

Though it took time to put into practice the declared policy change of disaster risk management in Ethiopia, after 1993 some of the prevention and the preparedness measures started taking place with the involvement of all line ministries and communities. Among these changes was the early warning system and assessment tool developed in 1977 being modified through time in order to make it serve the intended purpose 25 . The previous early warning system was focusing on the food security, rainfall, and water availability status; however, the modified one was made to be relatively more comprehensive by including the nutritional status of the vulnerable groups of population such as children under-five years and pregnant women in disaster prone areas 25 , 29 . This early warning system is being conducted on monthly and quarterly basis and reports are transferred from the community to the federal Disaster Risk Reduction and Food Security Sector, which could enable timely action 30 . The other measures taken as part of preparedness and preventive phase of disaster risk management is ensuring the food availability at government stock and timely appeal and request for further aid 31 ,32 . In addition, beginning from year 2000 in all drought prone districts, periodic community based nutritional screening started and the outpatient therapeutic feeding programs were initiated in all health stations (replaced by health posts since 2003) and health centers so as to manage uncomplicated malnutrition cases at the outpatient level 24 ,33 ,34 . Furthermore, at health centers and hospitals, therapeutic feeding units were opened so as to treat severe and complicated malnutrition cases using the national guideline and treatment protocols 34 . There have been other interventions undertaken by the government of Ethiopia in order to mitigate drought disaster; these interventions are designing and implementing irrigation schemes in drought areas 35 . Creation of employment opportunities to reduce vulnerability in their localities of those drought affected areas as part and parcel of mainstreaming the risk management interventions into the long term development programs are also undertaken 27 . On the other hand, the health sector implemented a clear surveillance system to control the outbreaks and respond on time to any emergency. Particular emphasis was put on the drought and malnutrition stricken areas due to the fact that in most instances malnutrition has been followed by the outbreaks of diseases 1 ,33 . Looking at other hazards apart from drought and diseases epidemics, since 2006 following the floods disaster across many parts of the country an early warning system for flood has begun in collaboration with the metrological agency and information is being conveyed to the inhabitants of the flood areas using mass-media and the disaster risk reduction and food security sector has been activating itself for effective preparedness and response to disaster 36 . In general, since changing the policy on disaster risk management although drought and disease epidemic disasters are happening more frequently, the number of deaths have been reduced dramatically compared with the early periods of disasters (1974, 1984, 1989). This could be due to the lesson learned from the previous disasters and effective preparedness and response actions under taken by the government and partners.

The disaster management system seems to be comprehensive in its approach and the policy changes created a conducive environment for change in practice of disaster risk management, specifically related to drought. The government has acknowledged that natural disasters from other hazards such as floods are emerging more frequently and their intensity is even becoming higher than that of drought. Lack of multi-hazard approach policy and strategy is one of the major weaknesses, as stated by the government recently. Cognizant of the challenge, at policy and strategy level this appears to be addressed in general terms 28 . However, still there is no clear and specific policy and strategy pertaining to these hazards apart from drought and when any disaster happens, the intervention being directed by government and partners is “reactive” than “pro-active”. The actions undertaken when flood happened in different parts of the country in 2006 is a case in point 15_. _In fact some lessons were drawn after 2006 widespread flooding events in the country, and an early warning activities have been initiated 36 . However, there is insufficient evidence that could show the structural and non-structural measures being under taken by government and non-governmental organizations to protect the vulnerable people.

**1.2 Ethiopian Health System’s Response to Disaster and Disaster Risk**

**1.2.1 Health System**

The health care system of Ethiopia has been modified over time from curative focused approach and highly centralized to more preventive centered and decentralized with few layers. This change is in agreement with the causes of morbidities and mortalities in the country as most of them are communicable diseases that could be averted through accessing primary health care services. Thus, under the current government, the Ethiopian health care system has been transformed from six-tier health care delivery system to four, and more recently to three-layers 6 ,33 ,37 ,38 ,39 . The current health delivery system has been built of Primary Health Care Unit (PHCU) from the bottom and intended for the maximum of 100, 000 people; the second layer from the bottom is the general hospital that serves up to 1.5 million people; and the third is specialized hospital serving around 5 million people 33 ,37_._

PHCU is formed by a primary hospital, a health center and five health posts. Primary hospital aimed to serve from 60,000 to 100, 000 people. Health center provides health service to 25, 000 people, and has five health posts as its catchment area in order to provide technical and administrative supports to health posts. A health post covers to the maximum of 5,000 rural people and staffed by two female health extension workers, who completed 10 years of education with additional one year of training in basic primary health care service, emphasized on preventive health care 33 ,40 .

The health care system under the leadership of the federal ministry of health is responsible for carrying out the routine and integrated surveillance. This is to immediately notify and investigate any suspected epidemic or outbreak of disease and take control measures in collaboration with other partners, mainly with World Health Organization. Therefore, at all levels from health posts and community to the Ethiopian federal ministry of health a well-functioning epidemic preparedness and control mechanism has been underway 1 ,41_._

**1.2.2 Health System’s Response**

When we see the general set up of the emergency medicine in Ethiopia, hospitals have outpatient emergency departments for the management of emergency cases (individuals visiting hospitals for emergency). The initial screening of patients done at emergency department and if any emergency surgery is needed, patients would be transferred to the respective department for surgical procedure or for advanced level of care. Basing on limited available evidence, there is no triage system in the emergency departments; and there is no out-of hospital emergency management system in Ethiopian health care system. During night time only one general practitioner and one nurse or “health assistance” would be assigned in the emergency department, and managing emergency cases as a result of mass causality during night could be very problematic. Moreover, the training of emergency medicine has been reported to be non-standardized in the country. There is no specific emergency medicine course in the training curricula of medical schools in Ethiopia; and the skills and knowledge of emergency medicine among health professionals in Ethiopia could not enable them to manage emergencies arising from natural and man-made disasters, particularly those causing huge number of injuries. 42 To fill such gap, some initiatives have been taken by the ministry of health and non-governmental organizations or partners. For instance, the Israeli government has supported the Ethiopian Ministry of Health in training of physicians in emergency medicine 42 ; Medicines Sans Frontiers (MSF) though its main operational mandate in Ethiopia is responding to emergency during outbreaks of diseases or disaster, it has taken initiatives to strengthen the hospitals’ capacity through training of general practitioners in emergency surgery 43 . The Ethiopian federal ministry of health in collaboration with ministry of education began offering Integrated Emergency Surgery Officer (IESO) training at masters level (three years training in integrated emergency surgery) beginning from 2009. By the end of 2011, totally 252 health officers (BSC holders with clinical and public health training) were enrolled for this program in five public universities 44 . This program has been initiated to increase the trained human resource in emergency surgery at hospitals and health centers. Though this program has not been directly related to emergency management arising from disasters, it has a significant contribution in building the public health facilities with trained man power in the area of emergency.

Similar to the disaster risk management system of Ethiopia, the health system’s response to emergencies arising from disasters came into being with series of drought disaster events in the country. Thus, reforms of disaster risk management policies and strategies in Ethiopia have paved the way for the involvement of the health sector in all phases of disaster management. Initially with the former RRC, the health sector role was not clearly stated and through time as a result of experience gained from responding to disasters, participation of all concerned sectors and preparedness for disaster was necessitated and included in subsequent reforms of policies and strategies 19 ,20 . Change in policies and strategies of disaster risk management enabled health system to take some initiatives and develop its own guidelines and strategies of preparedness for emergencies and response to disasters 1 ,34 . One of those initiatives being under taken under the leadership of Ethiopian ministry of health is the “Community Based Nutrition” in drought affected areas. Nutritional assessment among children under-two years is regularly undertaken and malnutrition suspected cases are referred to health facilities and health posts 33 . The second program that was adopted due to drought and malnutrition is therapeutic feeding program. In drought prone areas, ministry of health of Ethiopia in partnership with UNICEF and other International Non-governmental Organizations (INGOs) established an outpatient therapeutic feeding program at health posts (community based), at health centers and hospitals. While the management of malnutrition at the health posts level is limited to severe and uncomplicated cases, at health centers and hospitals the therapeutic feeding units have been established to manage severe and complicated malnutrition cases 34 . At health posts, which are the lowest level health units operating close to the community, severe and uncomplicated malnutrition cases are managed at outpatient department level and if cases failed to respond to treatments given, they would be referred to health centers. In addition, health posts have duty- bound responsibility to refer the severe complicated malnutrition cases to health centers and notify health centers any malnutrition and its associated outbreaks existence in their respective localities. At health centers and hospitals, besides the management of malnutrition patients at outpatient department level, they manage severe complicated cases by admitting to the therapeutic feeding units. Overall, using a national guideline, malnutrition cases from moderate to severe-complicated cases are being managed at different levels of health facilities and at the community level (health posts). The expansion of the therapeutic feeding program to different health facilities and health posts and to different regions or areas were step by step. At early stages of implementation of the program, its effectiveness could be below the expected level due lack of experience and trained manpower. For instance, in 2006 a qualitative study conducted in Borana drought and emergency (southern part of Ethiopia) and it pointed out that the health facilities were unprepared in terms of human power and therapeutic feeding items, and the surveillance system was not well functioning in order to respond to the emergency in the area 45 .

The therapeutic feeding programs have been proved to be cost effective in saving lives and the cure rates have been reported to be high in most studies conducted 46 ,47 ,48 . The study conducted in 2002 in the southern part of Ethiopia reported the recovery rate of 85% 46 . Another study conducted in 2006 in Southern, Northern and Eastern part of Ethiopia revealed the recovery rate of 79.4% 47 ; while a recently conducted study in 2012 in Northern part of Ethiopia shows a recovery rate of 62% 48 . According to the national annual reports, a cure rate from severe acute malnutrition among cases treated at health facilities and health posts in all drought prone areas has been 82%, 85%, and 86% for the year ending 20011, 20012, and 2013, respectively 16 ,49 . In all these studies and annual government reports, default due to unknown reasons from the treatment has been contributing 3.7% to 14 % of the total treatment outcomes.

Responding to disease outbreaks or epidemics is one of the responsibilities of the health system in Ethiopia. As part of the routine surveillance system, federal Ethiopian ministry of health has adopted World Health Organization’s strategy of surveillance system for developing countries, commonly known as “integrated surveillance system”. However, in 2012 the national guideline was prepared to make the health sectors approach in responding to disaster and emergency a “multi-hazards risk management approach”. This guideline states that besides responding to natural and man-made disasters, 20 selected diseases that are considered as of public health importance to be under routine surveillance system. Of these 20 diseases, 13 of them are required to be reported immediately while 7 of them should be notified every week 1_. _Some of these diseases included here such as measles, meningococcal meningitis, malaria, dysentery, cholera, and severe malnutrition are secondary to drought and floods disaster events. The existence of one case of polio, anthrax, cholera, avian human influenza, guinea worm or dracunculiasis, neonatal tetanus, pandemic influenza, rabies, smallpox, severe acute respiratory syndrome (SARS), viral hemorrhagic fever, and yellow fever are considered and handled as epidemic and their reporting from any health facility need to be done immediately. Diseases such as dysentery, malaria, meningococcal meningitis, relapsing fever, severe malnutrition, typhoid fever and typhus are reported on weekly basis, and declaring of epidemic for these diseases is based on the prior level of disease burden in a specific location 1 ,16 ,33 . Thus, when any epidemic is declared for a particular disease, the health system responds as an emergency health situation. Overall, the epidemic control system seems improving from year to year. However, in some cases the diseases status looks stable with little change. For instance, the average national case fatality rate of measles of 2011 was 0.5%, for 2013 was 0.4% and the average national case fatality rate of meningococcal meningitis for the year ending 2011 was 2.9%, and while for the year ending 2013 it was 2.5% 16 ,48 . In the year 2013 in the southern part of Ethiopia in two regions (Oromia and Southern Nations and Nationalities/SNNP), meningococcal meningitis outbreak was reported, control and preventive measures were taken by the government in collaboration with non-governmental organizations 16 .

The health system’s role with regard to floods, volcanic eruption, and other man-made hazards in terms of prevention, preparedness, mitigation, response and recovery has not been clearly stated in the strategy (guideline) of ministry of health or other government’s disaster risk management policies and strategies. Hence, there is little evidence of change in practice of the health sector in averting these hazards, apart from the declaration of policy. For example, in 2008 a study conducted in western part of Ethiopia in flood prone area revealed that the health facilities were unprepared for flooding and training was not given to health professionals; an early warning was not given to vulnerable residents in the area; materials necessary for emergency such as boats and life jackets were unavailable; there were no trained swimmers; and disaster prevention and preparedness agency in the area had no medicines to respond to epidemics after flooding 15 .

**1.2.3 Health System's Relationships with Partners**

During disaster and emergency, the health system is responding in synergy with other sectors and in collaboration with donor agencies and INGOs. Several INGOs began operating in Ethiopia in 1974 drought in order to respond to the then catastrophic humanitarian situation of northern part of the country. Ever since then, the roles and operational geographical areas of INGOs have been determined by the Disaster Prevention and Preparedness Commission, which has been recently changed and called Disaster Risk management and Food Security Sector 50 . Therefore, Ethiopia’s health system has been benefiting a lot from foreign aid/donors and INGOs in terms of direct financial support for financing the health care system 50 ,51 ,52 ; technical support in policy, strategy formulations, evaluation of emergency responses 1 ,20 ,27 ,53 ; training of human power in epidemic control and emergency response 42 ,43 ,50 ,54 ,55 ; and capacity building of health facilities with medical supplies, essential drugs and supplies mainly for malnutrition, measles, meningococcal meningitis, malaria, dysentery, and other common childhood and neonatal illnesses 56 ,57 ,58 . Its worth mentioning that, INGOs have been highly involved in designing the national malnutrition management protocol, and initiating the therapeutic feeding programs in drought prone areas of health facilities and posts. INGOs and UN agencies have a paramount importance role in assisting the ministry of health technically and financially in training of health workers in malnutrition management at outpatient departments and inpatient therapeutic feeding units, Integrated Management of Neonatal and Child-hood Illnesses (IMNCI), and surveillance system and responding to emergencies in the country 34 ,58 . This support is rendered through the government system to ensure sustainability of the program and integration of new initiatives into the health system. However, when a disaster situation and emergency is severe and appears to be overwhelming the health system, the INGOs are called on by the government to offer emergency medical services (to act even out of their normal operational areas) 24 . At the field level, they implement “emergency relief programmes” till the situation of epidemics become under control 46 . For example, when the severe drought occurred in Borana zone of Oromia region (southern Ethiopia) in 1984, and in Gode district of Somali region in 2000, INGOs were made to directly support the government health system at field level in responding to widespread cases of malnutrition and measles outbreaks 8 ,10 . Overall, INGOs and donors are playing a crucial role in the containment of emergencies due to drought disaster and outbreaks of diseases. However, as indicated previously INGOs and their practical roles in supporting the health system are minimal with regard to floods, earthquake, volcanic eruptions, man-made and other hazards.

In general, a lot of lessons have been learnt from disasters over time and some have been incorporated into the policy and ultimately resulted in change in emergency medical practice such as therapeutic feeding programs in health facilities and investigation and control of outbreaks. Since the recent years, due to the widespread existence of natural and man-made disasters in the country, the public health emergency guideline 1 has been developed and risk assessment, multi-hazard approach, participation of all sectors and INGOs are considered as the corner stone priority approach of the health sector toward disaster and disaster risk management. However, apart from the drought and disease epidemics little evidence exists in proving whether lessons learnt were put into practice.

**1.3 Strengths and Limitations of Disaster Risk Management in Health System**

****In the course of implementing the disaster risk management programs in Ethiopia, there are some strong points. Firstly, the disaster risk management system of Ethiopia that used to focus on response and recovery, transformed itself to include mitigation, prevention, preparedness components, and currently the system is comprehensive, particularly for drought 27 ,28 . Secondly, as the task of disaster management requires multisectorial approach, all line ministries including ministry of health have been involved from federal to the district level and other partners like non-governmental organizations are part of policy formulation and implementation 27 . Thirdly, some emergency programs such as the malnutrition have a clear guideline to be implemented at all levels of health facilities and at the community level in drought prone areas 34 . Fourth, for drought the government has linked the program with the other development programs in order to tackle the challenge in the long term 27 . Finally, apart from collaborating with Disaster Risk Management and Food Security Sector for all natural and man-made disasters, the ministry of health recently developed a guideline for the management of public health emergencies and 20 diseases have been identified to be under surveillance at all levels of health care system and at community level 1 ,16 ,33 . Hence, these are the major strengths of the disaster management system within the Ethiopian health sector.

On the contrary, the disaster risk management system has some limitations which include lack of detailed policies and strategies for other hazards such as flooding, earthquake, landslides, and volcanic eruptions. Moreover, the responsibilities of ministry of health is not clearly stated in terms of policy and strategy to mitigate, prevent, and get prepared for hazards that include flood, earthquake, landslides, and volcanic eruptions.

## CONCLUSIONS

The disaster risk management system in Ethiopia has been changed over time from response, recovery and rehabilitation to more comprehensive by incorporating additional components such as mitigation, prevention and preparedness for a disaster. Due to its history, the focus of disaster management till recently is focusing on drought though the impacts of other disasters are also prevailing. The health care system of Ethiopia also evolved over time from curative centered approach to preventive, and the current health care system has three layers that include primary health care unit, general and specialized hospitals from bottom to top, respectively. The health sector has taken initiatives for management of disasters related to drought and its impacts in collaboration with Disaster Risk Management and Food Security Sector. In addition, the ministry of health has developed a guideline by which it manages the public health epidemics and 20 diseases have been identified to be under regular surveillance. However, the Disaster Risk Management and Food Security and Ministry of Health need to put more efforts to address the impacts of other disasters.

## COMPETING INTEREST

The authors have declared that no conflicts of interest exist.
